# Effects of ACEIs Versus ARBs on Proteinuria or Albuminuria in Primary Hypertension: A Meta-Analysis of Randomized Trials

**DOI:** 10.1097/MD.0000000000001560

**Published:** 2015-10-02

**Authors:** Rui Xu, Shanmei Sun, Yan Huo, Lin Yun, Shuai Huang, Guohua Li, Suhua Yan

**Affiliations:** From the Department of Cardiology, Shandong Provincial Qianfoshan Hospital, Shandong University (RX, SS, YH, LY, SH, GL, SY); and Shandong University of Traditional Chinese Medicine, Jinan, P.R. China (SS, YH, SH).

## Abstract

Although angiotensin-converting enzyme inhibitors (ACEIs) and angiotensin receptor blockers (ARBs) belong to a family of therapies that block the renin–angiotensin system and are suggested to improve proteinuria/albuminuria, it is unclear which is more effective.

To compare the effects of ACEIs and ARBs on proteinuria in primary hypertension by performing a meta-analysis covering randomized controlled trials (RCTs).

We systematically searched MEDLINE, EMBASE, and the Cochrane Central Register of Controlled Trials from January 1990 to November 2014. Eligible studies were RCTs of ACEI therapy versus ARB therapy that reported the albumin excretion rate (AER), albumin (Alb), and urinary albumin excretion (UAE) as outcomes.

Seventeen RCTs, including 17,951 patients (without limit of race, age, or sex) with a mean duration of 62.6 weeks, were included. Pooled analysis suggested that ACEIs and ARBs showed no significant differences in AER/Alb/UAE/24-h urine protein/24-h urine total protein in a comparison of 10 trials (SMD 0.09; 95% CI –0.18–0.36; *P* = 0.52). No significant differences were observed in urinary protein/creatinine ratio (UPCR)/urinary albumin/creatinine ratio (UACR), or albumin/creatinine ratio (ACR) in 7 trials (SMD 0.15; 95% CI –1.88–2.19; *P* = 0.88). The total outcome of ACEIs and ARBs also showed no significant difference (SMD 0.13; 95% CI –1.03–1.29; *P* = 0.83). The efficacies of ACEIs and ARBs in controlling blood pressure as a secondary indicator were also similar (SMD –0.50; 95% CI –1.58–0.58; *P* = 0.37).

Based on a meta-analysis of 17 randomized controlled trials including 17,951 patients, we found that ACEIs and ARBs can reduce urine protein levels, improve blood pressure, and were similarly effective in terms of reducing urinary protein excretion.

## INTRODUCTION

Primary hypertension, one of the most prevalent and hazardous causes of cardiovascular disease can also lead to renal damage. Hypertension is associated with chronic kidney disease (CKD) and can also lead to end stage renal disease (ESRD), not only the person of African ancestry.^1–3^ Activation of the renin–angiotensin–aldosterone system (RAAS), especially angiotensin II, plays an important role in its hemodynamic pathophysiology. The 8th Joint National Committee (JNC8)^3^ reported new guidelines for the management of high blood pressure, and recommended that in the population aged >18 years with CKD, initial antihypertensive treatment should include angiotensin-converting enzyme inhibitors (ACEIs) or Ang-II receptor blockers (ARBs) to improve kidney outcomes. As agents for blocking of the renin–angiotensin system, ACEIs and ARBs have equal efficacy in terms of controlling blood pressure and improving renal function. Although some related analyses indicated a small difference in efficacy between ACEIs and ARBs, the investigations were not comprehensive, and little evidence is available regarding which is more effective in treating proteinuria.

In this study, we performed a meta-analysis of the extant trials, assessing renal outcomes of hypertensive patients treated with either ACEIs or ARBs.

## METHODS

The guidelines of the Preferred Reporting Items for Systematic Reviews and Meta-Analyses (PRISMA)^4^ were followed in all the phases of the study, that is, during the design, implementation, analysis, and reporting. We performed a comprehensive and systematic search of MEDLINE, EMBASE, and the Cochrane Central Register of Controlled Trials using Web-based search engines (PubMed, OVID), China Biology Medicine (CBM), China National Knowledge Infrastructure (CNKI), and the Wanfang Data, from January 1990 to November 2014. The search was restricted to randomized controlled trials (RCTs) of ACEI versus ARB therapy in humans published in peer-reviewed journals; all included studies were required to report the albumin excretion rate (AER), albumin (Alb) level, and urinary albumin excretion (UAE) level as outcomes. If some data were unavailable, or if local libraries were unable to retrieve the full paper, the authors were contacted via e-mail. No language restriction was applied; non-English-language studies were translated by native speakers experienced in the health field. We reviewed the reference lists of the articles and original studies identified by the electronic search for other potentially eligible articles. If multiple publications addressed the same dataset, the most recent complete report was included. All analyses were based on previous published studies; thus no ethical approval and patient consent are required.

### Study Selection and Data Extraction

**FIGURE 1:**
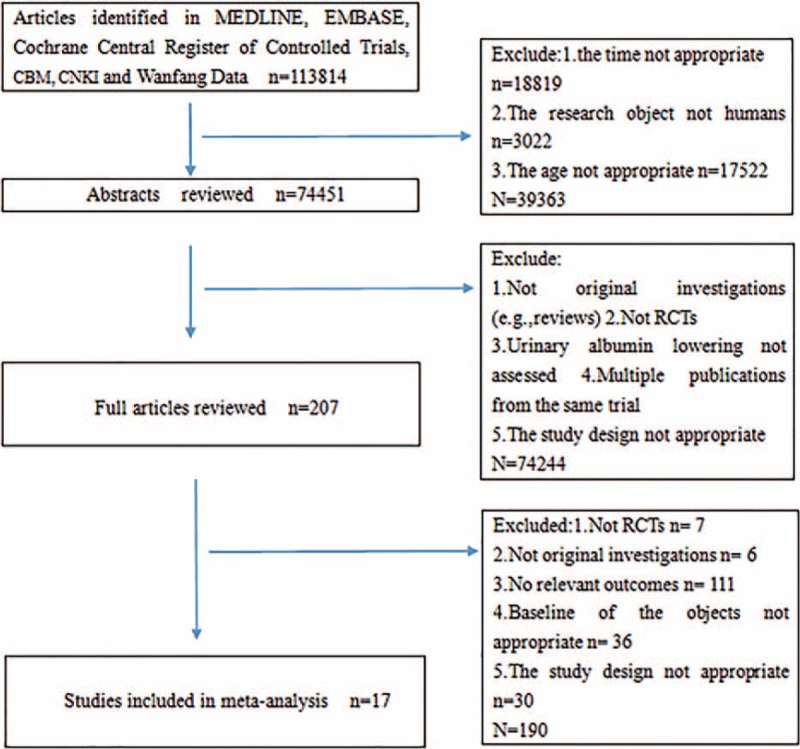
Flowchart of study selection. CBM = China Biology Medicine; CNKI = China National Knowledge Infrastructure; RCT = randomized controlled trial.

Two authors searched the data independently. Disagreements were resolved by discussion with a third party until a consensus was reached. For studies to be included they had to fulfill the following criteria: the design was a prospective randomized controlled clinical trial; it was published between January 1990 and November 2014; the population was primary hypertensive with or without diabetes; patients were randomly assigned to ACEIs or ARBs; and outcomes included urine protein excretion (UPE), UAE, urinary protein/creatinine ratio (UPCR), or urinary albumin/creatinine ratio (UACR) levels. Data regarding detailed inclusion criteria, the research object, experimental measures, duration of follow-up, and UPE/UAE/UPCR/UACR levels were extracted (as available) from each study. Research was eliminated if it included primary diseases of the kidney system (including renal transplantation), CKD (eliminated as CKD can also cause proteinuria, including glomerular nephritis, nephrotic syndrome, IgA nephropathy, membranous nephropathy, and systemic lupus with lupus nephropathy), type I diabetes, diabetic nephropathy, and secondary hypertension. Other articles that were secondary research data, not clinical trials, were incomplete, or had obvious errors excluded (Fig. 1).

### Quality Assessment

The quality of the publications was evaluated independently by 2 researchers, and in the case of disagreements, decisions were resolved through discussion. The criteria used for quality assessment related to whether the trial involved a double-blind patient assignment, whether complete outcome data were presented, whether there was any evidence of selective outcome reporting, or other sources of bias, as recommended by the Jadad rating scale. We classified studies that had a high or unclear risk of bias for any of the first 3 components to be of low quality.

### Statistical Analysis

Statistical analyses were performed according to the recommendations of the Cochrane Collaboration and using Review Manager (RevMan), version 5.2 (Cochrane Collaboration, 2013). Continuous variables were combined to give values of mean difference (MD) or standardized mean difference (SMD) and 95% confidence intervals (CI). If the research data were in the form of binary classification variables, we used the odds ratio (OR), relative risk (RR), or dangerous poor (risk difference, RD) to combine the statistics and 95% CI. The heterogeneity among all studies was analyzed using the *χ*^2^ test. *I*^2^ was the proportion of total variation observed between the trials attributable to the differences between the trials rather than to sampling error (chance), and we considered *I*^2^ < 25% as representing low heterogeneity and *I*^2^ > 75% as representing high heterogeneity. If there was low heterogeneity (*P* ≥ 0.1 and *I*^2^ ≤ 50%), the fixed-effect model was used. If there was high heterogeneity (*P* < 0.1 or *I*^2^ > 75%), the clinical heterogeneity was analyzed. If the cause was not clear, a random effects model was used.^5^ If the results were measured using the same units of weights and measures, the weighted mean differences (WMD) were selected; if a different measurement unit was used, the SMD was used. Results were calculated by MD or SMD and 95% CI. Analysis was stratified by patients with an outcome of “AER/Alb/UAE/24-h urine protein/24-h urine total protein” or “UPCR/UACR/ACR/protein to creatinine.” Analysis was also performed to evaluate the efficacies of ACEIs and ARBs in terms of controlling blood pressure. Sensitivity analysis was performed by omitting studies based on quality assessment and checking the consistency of the overall effect estimate. Publication bias was evaluated using a funnel plot.

## RESULTS

### Total Results

In total, our meta-analysis selected 17 randomized controlled clinical trials including data on 17,951 patients randomized to therapy with ACEIs (n = 9036) or ARBs (n = 8915). The trial design, follow-up time, proportion, and dose of medication, and the Jadad grading are shown in Table 1. In 1 study, a section of the trials was divided into 2 or 3 subgroups according to follow-up, indicated by A, B, and C. Ten studies investigated renal damage using an index such as “AER/Alb/UAE/24-h urine protein/24-h urine total protein,” and others with the index “UPCR/UACR/ACR/protein to creatinine.” The baseline, follow-up, and changes in the indices are summarized in Table 2 . To facilitate statistical analysis, a portion of the data in Table 2  was transformed from median/mean (min–max) to mean ± standard deviation, according to Hozo et al.^6^ The standard deviation of the ONTARGET study^14^ data was calculated according to the Cochrane Handbook for Systematic Reviews of Interventions, version 5.1.0 (updated March 2011).^7^ To expand our data, the DETAIL^25^ data were also calculated according to Takagi.^8^ According to the Clinical Practice Guidelines of CKD,^26^ if the UPCR is expressed in mg/mg the value obtained is approximately the same as the number of g/24 h of urinary protein excretion. Therefore, we unified the units of AER (mg/24 h) and ACR (mg/g) (Table 2 ) to enable subgroup analysis. To compare the efficacy in terms of controlling blood pressure, we selected the baseline and follow-up systolic and diastolic blood pressure from 12 of the 17 trials (Table 3).

**TABLE 1:**
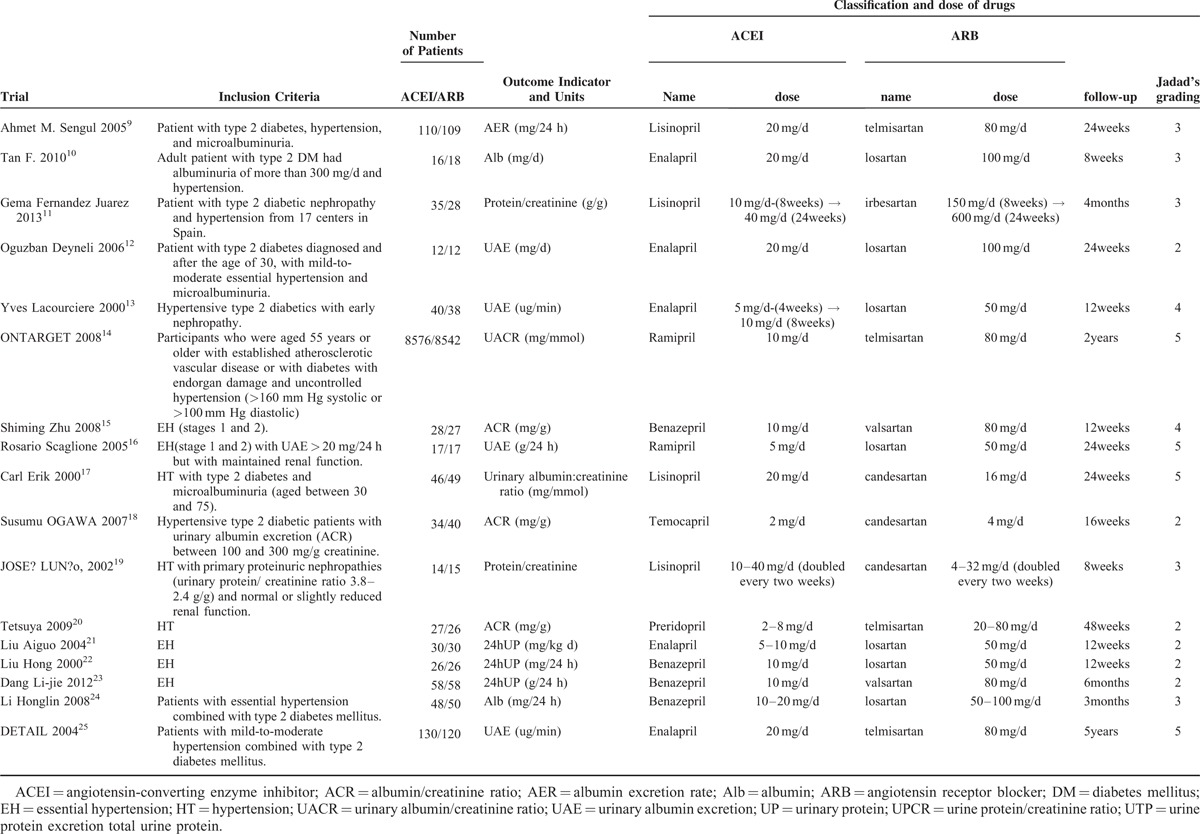
Trial Design and Estimate

**TABLE 2:**
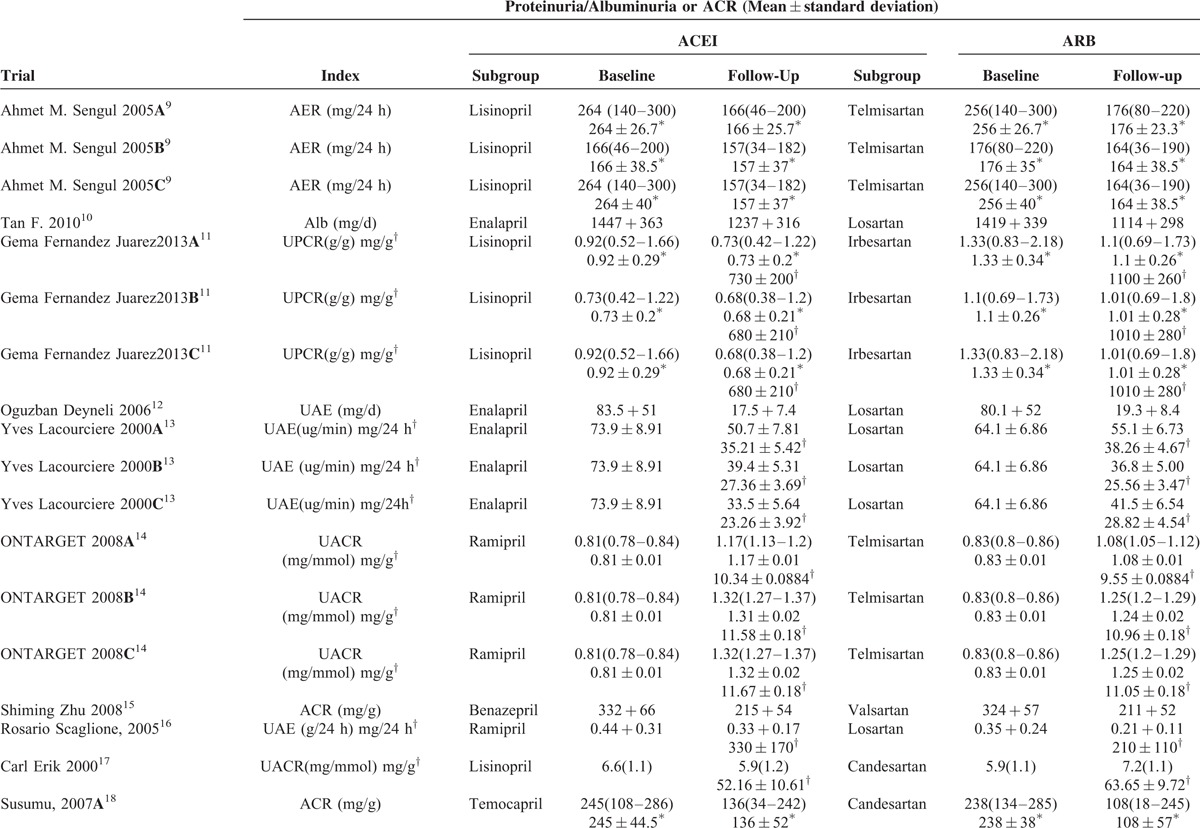
Baseline and Follow-Up Proteinuria/Albuminuria or ACR and Change of Them

**TABLE 2 (Continued):**
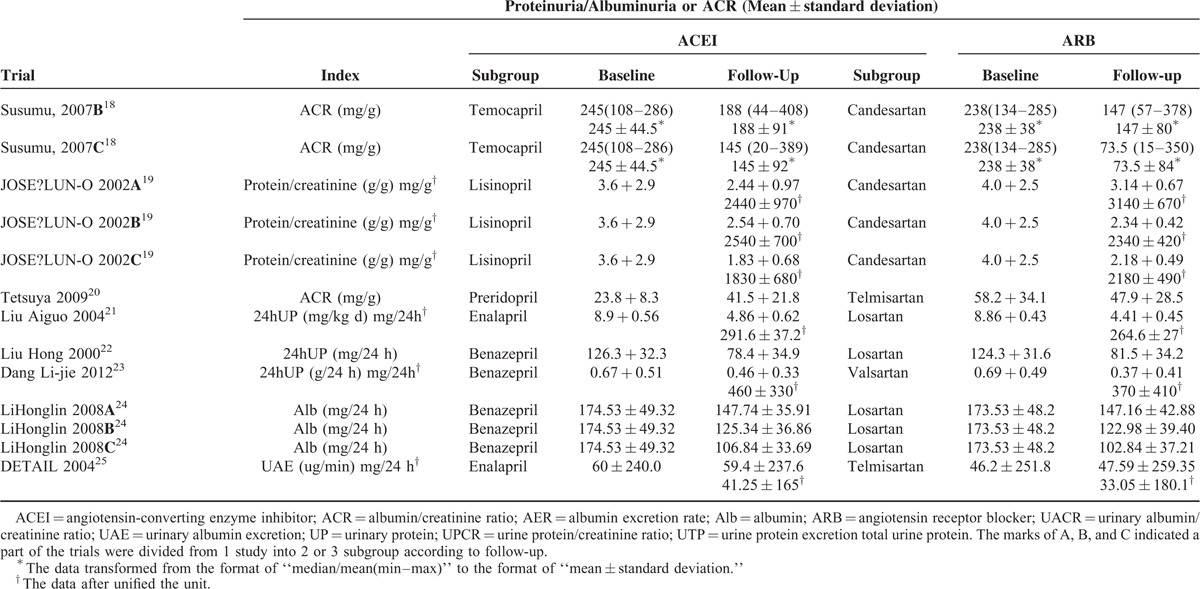
Baseline and Follow-Up Proteinuria/Albuminuria or ACR and Change of Them

**TABLE 3:**
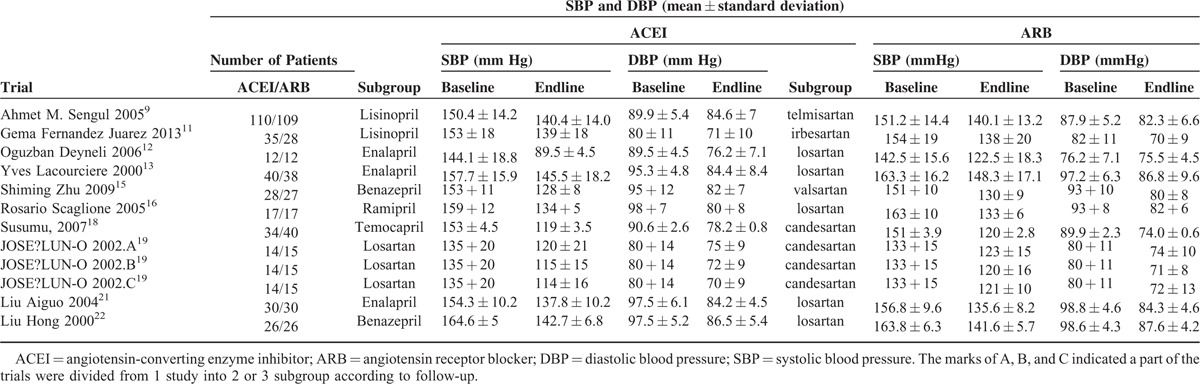
Baseline and Follow-Up Blood Pressure and Change of Blood Pressure

**FIGURE 2:**
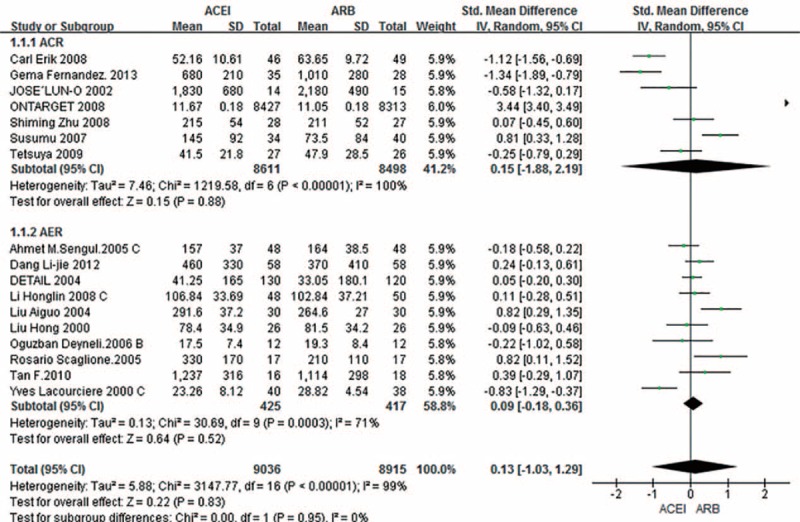
Subgroup analysis based on outcome in AER and ACR among patients randomized to ACEI versus ARB. ACEI = angiotensin-converting enzyme inhibitors; ACR = albumin/creatinine ratio; AER = albumin excretion rate; ARB = angiotensin receptor blockers; CI = confidence interval; SD = standard deviation; SMD = standardized mean difference.

Pooled analysis of “UPCR/UACR/ACR” from the 7 studies^11,14,15,17–20^ suggested marked heterogeneity (*I*^2^ = 100%); therefore, the random effects model was used. The outcome showed no significant differences in AER/Alb/UAE/24-h urine protein/24-h urine total protein in 10 trials (SMD 0.09; 95% CI –0.18–0.36; *P* = 0.52) and “UPCR/UACR/ACR” in 7 trials (SMD 0.15; 95% CI –1.88–2.19; *P* = 0.88) (Fig. 2).

**FIGURE 3:**
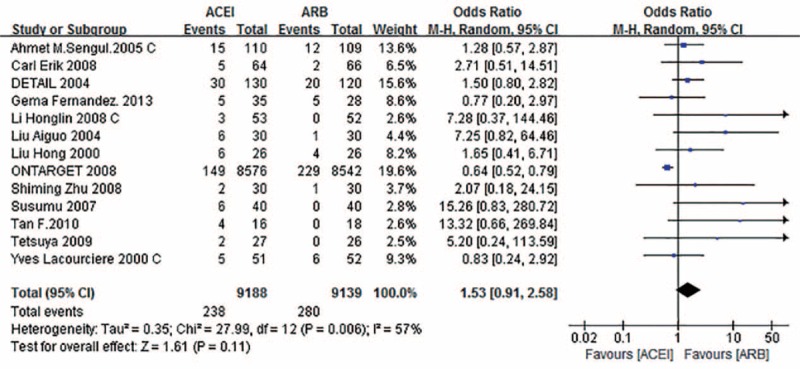
Forest graph of the meta-analysis of the effect of ACEI versus ARB in adverse reaction. ACEI = angiotensin-converting enzyme inhibitor; ARB = angiotensin receptor blocker; CI = confidence interval.

The rates of adverse reactions, including cough, headache, and stomachache, were somewhat lower when ARBs were given rather than ACEIs (OR 1.53; 95% CI 0.91–2.58; *P* = 0.11) (Fig. 3); however, the difference was not statistically significant.

**FIGURE 4:**
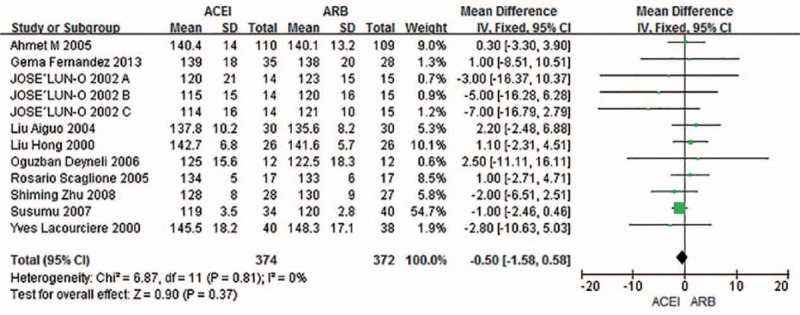
Forest graph of outcome in systolic blood pressure of 12 trials selected from all the 17 trials. ACEI = angiotensin-converting enzyme inhibitor; ARB = angiotensin receptor blocker; CI = confidence interval; SD = standard deviation.

Moreover, the efficacy of ACEIs and ARBs in terms of controlling systolic blood pressure was similar (MD –0.50; 95% CI –1.58–0.58; *P* = 0.37) (Fig. 4).

### Subgroup Analysis

**FIGURE 5:**
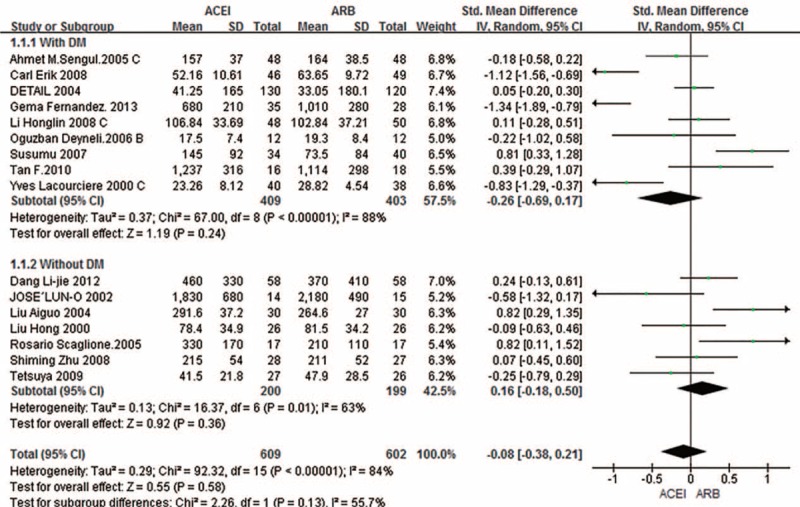
Subgroup analysis based on patients with or without diabetes. ACEI = angiotensin-converting enzyme inhibitor; ARB = angiotensin receptor blocker; CI = confidence interval; DM = diabetes mellitus; SD = standard deviation; SMD = standardized mean difference.

**FIGURE 6:**
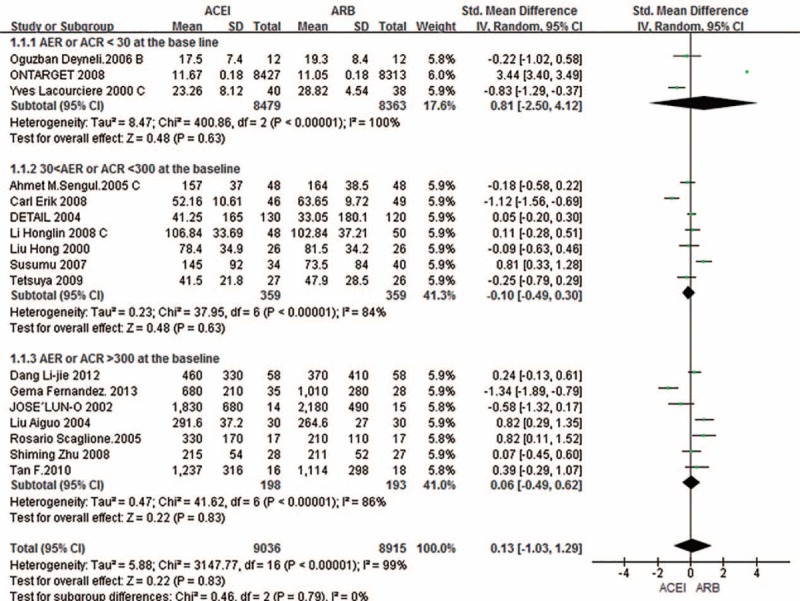
Subgroup analysis based on the baseline of AER or ACR. ACEI = angiotensin-converting enzyme inhibitor; ACR = albumin/creatinine ratio; AER = albumin excretion rate; ARB = angiotensin receptor blocker; CI = confidence interval; SD = standard deviation; SMD = standardized mean difference.

Subgroup analysis indicated considerable heterogeneity. To address this heterogeneity, 4 subgroup analyses were performed to assess the effects of patients with or without diabetes; ACEIs and ARBs had similar contribution (SMD –0.08; 95% CI –0.38–0.21; *P* = 0.58) (Fig. 5). We excluded the ONTARGET study as the number of diabetic patients enrolled was unclear. As shown in Fig. 6, when 30 and 300 mg/24 h or mg/g were taken as the boundaries for normal proteinuria, microproteinuria, and proteinuria, respectively, subgroup analysis was used to assess the effects of baseline AER and ACR levels, and no difference was evident when the ACEIs and ARBs were compared among the 3 subgroups (SMD 0.13; 95% CI –1.03–1.29; *P* = 0.83). Finally, in subgroup analysis of the effects of follow-up duration, there were no differences (SMD 0.36; 95% CI –0.54–1.25; *P* = 0.43) when 6 months was used as the cutoff for treatment duration. With regard to the effect of dosage, we defined the small dose as the initial dose of the drugs, and the large dose was at least double doses of the initial dose. However, there was no clear difference related to whether a small (SMD 0.25; 95% CI –0.21–0.7; *P* = 0.28) or large dose (SMD 0.03; 95% CI –1.57–1.64; *P* = 0.97) was administered. As a supplement, we performed the same analysis in primary hypertension patients with diabetes, and there was also no difference in outcome (SMD –0.26; 95% CI –0.69–0.17; *P* = 0.24).

We were unable to remove the heterogeneity from subgroup analysis, but only the ONTARGET study patients had normal urine protein at baseline, and the number of patients was large, which would bias the heterogeneity. Removal of the ONTARGET study from the ACR analysis resulted in a reduced *I*^2^ value (*I*^2^ = 83%).

### Publication Bias and Sensitivity Analysis

**FIGURE 7:**
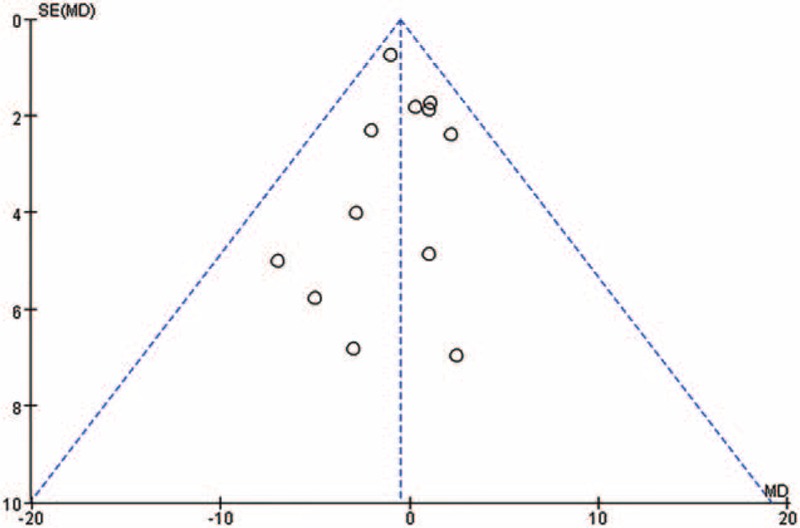
Funnel plot of the systolic blood pressure of 12 trials selected from all the 17 trials shows no significant evidence of asymmetry. MD = mean difference.

According to the retrieval strategy, the selected studies showed no publication bias. This was also the case for systolic blood pressure (Fig. 7).

Based on quality assessment, 11 studies were considered to have a low risk of bias, while the remaining studies exhibited a high risk. To assess bias, we performed analysis of trial sensitivity. After excluding data that had a large bias, the outcome suggested no significant between-drug difference (SMD 0.08; 95% CI –1.45–1.69; *P* = 0.92).

## DISCUSSION

The concept of using albuminuria as a surrogate marker for CKD progression and CVD outcomes is widely accepted, with the reduction of urine albumin levels often being regarded as a target for therapy.^27,28^ Many studies have shown that ACEIs and ARBs can reduce urine protein levels, which is considered to be independent of their kidney protection function, separate from antihypertension. The reduction in proteinuria is superior to that induced by other antihypertensive drugs.^29^ The rationale for using ARBs, unlike ACEIs, was that they block the angiotensin II type 1 (AT1) receptor, preventing the effects of the ACE pathway. However, the 2 pathways produce the same effect and induce a more complete inhibition of RAAS, resulting in increased endothelial nitric oxide production.^30^

According to our research, treatment with ARBs and ACEIs had similar efficacy in terms of improving blood pressure and preventing progression of proteinuria/albuminuria in primary hypertension. A recent meta-analysis^31^ showed that ARBs were more effective than ACEIs in reducing proteinuria in hypertensive patients. However, this investigation was not comprehensive and showed clear heterogeneity (*I*^2^ = 96%). Another meta-analysis comparing the effects of monotherapy and combination therapy with inhibitors of the renin angiotensin system on proteinuria^32^ concluded that the ARBs reduced proteinuria, independent of the degree of proteinuria and underlying disease. The magnitude of effect was similar regardless of whether the comparator was placebo or a calcium-channel blocker. A previous meta-analysis compared the effects of the ARB telmisartan on proteinuria or albuminuria with other ARBs, ACEIs, other antihypertensive drug therapies, placebo, or no medication.^8^ Their meta-analysis suggested that telmisartan therapy was likely to improve proteinuria/albuminuria over the short- to medium term and to prevent the progression of proteinuria/albuminuria over the medium term.

This meta-analysis, which included 17,951 patients, sought to evaluate the effects of RAAS inhibitors on proteinuria or albuminuria in primary hypertension. The experiments were selected to avoid the influence between the drugs. Furthermore, all patients underwent at least 2 weeks of placebo treatment, making the experimental design more scientific and rational. Only ACEIs and ARBs had a direct effect on urine protein levels. Overall, the results showed an equivalent effect associated with the class of RAAS inhibitors over a mean duration of 62.6 weeks. Furthermore, it should be emphasized that this meta-analysis was designed to allow a head-to-head comparison of ACEIs and ARBs. Notably, our analysis incorporated various doses of RAAS inhibitors and varying durations of therapy. We performed subgroup analysis covering a variety of dosages and found no difference between the 2 drug classes or according to the dose used. However, the large number of drug classes precluded performance of subgroup analysis. In our meta-analysis, the ONTARGET study,^14^ which included 16,740 patients with a median 56-month follow-up, showed that the effect of telmisartan on major renal outcomes in people at high vascular risk was similar to that of ramipril. This finding was supported by almost all of the trials with reasonable follow-up, showing that the therapeutic effects of ACEIs and ARBs were equivalent. As the numbers of patients in the ONTARGET study dwarf those of all other studies combined, we ran data from the other 16 studies to examine whether there was any change. We found the Forest plot showed no offset. The meta-analysis showed that the 2 classes of therapies yielded the same improvement in proteinuria/albuminuria over the short to medium term and prevented the progression of proteinuria/albuminuria during the medium term in primary hypertension patients with proteinuria. Our findings are important as the analysis included a large number of patients. The findings are relevant to clinical practice, as they are based on data from well-designed randomized trials encompassing a broad population of patients with high blood pressure and (or) diabetes mellitus (DM), who were treated for proteinuria or albuminuria and who are representative of hypertensive patients seen in clinics.

Diabetes mellitus is associated with many macrovascular complications, especially with hypertension. Approximately 40% of patients with type 2 DM at the age of 45 years were hypertensive; the proportion increases to 60% by the age of 75 years.^33^ Both hypertension and DM markedly accelerated the progression of proteinuria/albuminuria. A recent meta-analysis^34^ of patients with DM investigating the effects of ACEIs and ARBs on all-cause mortality, cardiovascular (CV) death, and CV events found that ACEIs reduced all-cause mortality, CV mortality, and major CV events in patients with DM, whereas ARBs had no such beneficial effects. The highlight of our meta-analysis is the performance of subgroup analysis of individuals with DM, and found that ACEIs and ARBs had analogous effects in terms of improving the AER and ACR. However, the main source of heterogeneity was from the ACR, which was assessed in only 3 trials. Moreover, as stated, there was considerable variation in the urine protein levels. Performance of further large trials with long-term follow-up will decrease the heterogeneity of this subgroup. However, further investigations are required to determine the equivalence of AER and ACR in the assessment of urine protein in patients with DM. Other findings of our meta-analysis showed that ACEIs have a greater effect in hypertensive patients with DM and that ARBs have a greater effect in patients without DM; the effects trend more to ACEIs at the 6-month follow-up and trend to ARBs thereafter; however, these differences were not significant.

In addition, we assessed changes in systolic and diastolic blood pressure as a secondary indicator of the antihypertensive effects of the 2 types of drug. A previous meta-analysis in hypertensive patients^35^ showed that the overall reduction in all-cause mortality resulted almost completely from ACEIs; ARBs did not reduce mortality. We found that the effects of ACEIs and ARBs in terms of controlling blood pressure and improving proteinuria were similar. Therefore, in view of the high prevalence of hypertension in the general population, widespread use of ACEIs may control blood pressure to a certain extent, reducing the incidence of hypertensive nephropathy.

However, we realized that high heterogeneity, resulting mainly from ACR (including the ONTARGET study), was present. A subgroup analysis was performed to investigate whether potential effect modifiers could explain any of the heterogeneity in treatment effects between studies. We found that the main source of heterogeneity was the ONTARGET study, which investigated individuals with high vascular risk, and the participants’ initial urine protein levels were low, or in some cases negative. Furthermore, the heterogeneity was reduced by the removal of the ONTARGET study. Moreover, we performed a further subgroup analysis of the stage of proteinuria and using different follow-up times, and found a high degree of heterogeneity.

Several limitations of our analysis should be discussed. There was considerable variation among the study populations. For example, the research populations were not unified, and the dosages of the active and control drug, target proteinuria levels, and follow-up times differed. Moreover, in several studies patients had other concomitant conditions and had received background therapy. Although these did not reduce the generalizability of our results, they made it challenging to accurately estimate the effects of ACEIs and ARBs in terms of improving blood pressure and preventing progression of proteinuria/albuminuria.

## CONCLUSION

ACEIs and ARBs can reduce urine protein levels, resulting in an improvement in blood pressure. There was no significant difference between the 2 drug types in reducing urinary protein excretion in patients with primary hypertension. In summary, by improving proteinuria or albuminuria in patients with primary hypertension, especially those with DM, the widespread use of ACEIs may improve many clinical outcomes. However, our conclusions are not specific to patients with chronic kidney disease. This study highlights the need for further large, high-quality studies to enable more reliable conclusions to be drawn.
